# More than two decades since Abuja declaration: A way forward for ending AIDS as a public health threat by 2030

**DOI:** 10.4102/jphia.v16i1.1272

**Published:** 2025-06-04

**Authors:** Nebiyu Dereje, Mosoka P. Fallah, Raji Tajudeen, Marta M. Terefe, Ngashi Ngongo, Nicaise Ndembi, Jean Kaseya

**Affiliations:** 1 Africa Centres for Disease Control and Prevention, Addis Ababa, Ethiopia

**Keywords:** human immunodeficiency virus, acquired immunodeficiency syndrome, Abuja declaration, Africa, health financing

## Abstract

The Abuja Declaration, which was endorsed in 2001, was a hallmark of African leadership’s decision to prevent and control human immunodeficiency virus (HIV) and acquired immunodeficiency syndrome (AIDS) in Africa. Since this declaration, there have been several achievements recorded in the fight against HIV and AIDS. This includes increased domestic and international financing, ground-breaking innovations and discoveries for effective screening, diagnosis, and treatment of HIV and AIDS, targeted interventions to address mother-to-child transmission, and tailored and innovative approaches to prevent new HIV infections, particularly among the key and vulnerable populations. However, unaddressed challenges still require urgent and accelerated interventions to attain and sustain the set 95-95-95 Joint United Nations Programme on HIV and AIDS (UNAIDS) target. As we are near the 2030 landmark, revitalisation of the commitments made in the Abuja Declaration is essential. African countries must increase their domestic resources to address the inequities and improve access to essential HIV and AIDS prevention and response interventions, particularly for adolescent girls and young women, children, and vulnerable populations. Revitalisation of sex education, social protection, and revisiting in-country laws that negatively impact the HIV prevention and response efforts are more essential than ever before. There is a clear need for rededication of political and leadership will and commitment as we envision epidemic control of HIV and AIDS by 2030. Countries need to develop an action-oriented, targeted, and all-inclusive roadmap for HIV and AIDS epidemic control by 2030.

## Introduction

Cognisant of the benefits to the economy, saving lives and protecting the social well-being of the population, global leaders, and more importantly, African Heads of State, have made several commitments to respond to the human immunodeficiency virus (HIV) epidemic and end acquired immunodeficiency syndrome (AIDS) as a public health threat by 2030. The ‘Abuja Declaration’, endorsed by the African Union (AU) member states in 2001, was one of these commitments.^1^ The Abuja Declaration stipulated that the AU member states commit 15% of their annual national budget to the health sector and called upon donor countries to provide 0.7% of their gross national product (GNP) as official development assistance (ODA) to developing countries. The Declaration also highlighted the essentiality of intensive resource mobilisation, expansion of access to HIV treatment, care, and support, strengthening the health system, improving access to affordable drugs and technologies, and strengthening research and development (R&D) on HIV and AIDS.^1^

Since the Abuja Declaration, significant progress and achievements in HIV response in Africa have been made, including increased domestic and international financing, ground-breaking innovations and discoveries for effective screening, diagnosis, and treatment of HIV and AIDS, targeted interventions to address mother-to-child transmission, and tailored and innovative approaches to prevent new HIV infections, particularly among the key and vulnerable populations. However, unaddressed challenges still require urgent and accelerated interventions to attain and sustain the set 95-95-95 Joint United Nations Programme on HIV and AIDS (UNAIDS) target.^2^ In this article, we critically analyse the achievements, success stories, and challenges in HIV and AIDS response in Africa since the Abuja Declaration in the light of identifying ways forward for the envisioned target of ending AIDS as a public health threat by 2030.

After 5 years of the Abuja Declaration, in May 2006, the AU Heads of State convened under the theme ‘Universal Access to HIV and AIDS, TB and malaria Services by 2010’ and reaffirmed their commitment to ensure universal access to HIV, AIDS, tuberculosis (TB) and malaria services in Africa.^3^ The summit recognised marked progress in resource allocation, wherein one-third (33%) of AU member states have allocated about 10% of their national budget to the health sector. The summit ended with a call for accelerated actions for universal access to HIV and AIDS, TB, and malaria services by 2010 through rededication of leadership at national, regional, and continental levels to implement priorities stipulated in the Abuja Declaration. The summit also calls upon civil society organisations, private sectors, and the international community to make concerted efforts to fight against HIV and AIDS, tuberculosis, and malaria in Africa.^4^ In July 2013 (Abuja +12), the AU Heads of State met to review the 12 years’ progress – underscoring the need for ensuring ownership, accountability, and sustainability of HIV and AIDS, TB, and malaria services in Africa. The Abuja +12 summit recognised the significant progress made towards improving access to HIV prevention, treatment, social protection, care and support, resource mobilisation, strengthening service delivery, sustainable financing, governance, and leadership. More importantly, the summit also noted unmet needs regarding access to HIV prevention and treatment services among children and limited improvement in strengthening the health system.^5^

Notably, the 2012–2015 AU Roadmap on Shared Responsibility and Global Solidarity for AIDS, TB, and Malaria Response in Africa emphasised three critical actions: more diversified and sustainable financing, local production of medicines and regulatory harmonisation, and improved leadership, governance, and oversight.^6^

## Investment in health

**FIGURE 1:**
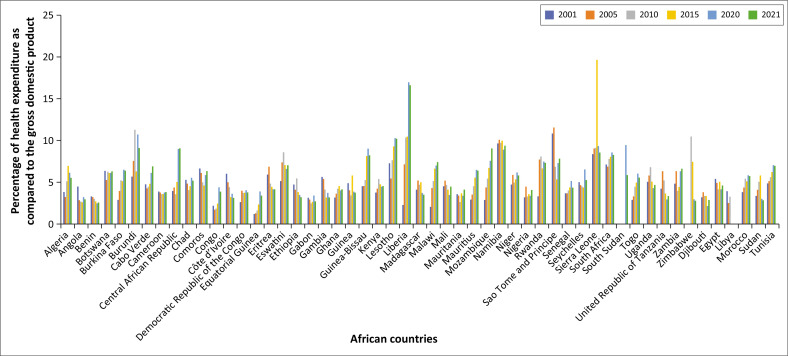
Current health expenditure (CHE) as % gross domestic product (GDP) of African countries, 2001–2021.

Twenty-three years after the Abuja Declaration, countries are struggling to meet the target of allocating 15% of their annual national budget to the health sector. Only about one-third (33%) of African countries have allocated about 10% of their national annual budget to the health sector (Figure 1).^7^ Countries failed to meet this target because of various reasons, such as competing priorities, limited fiscal space, debt obligations, corruption and resource mismanagement, a lack of accountability and proper governance, and donor dependence, among others.^8,9^

**FIGURE 2:**
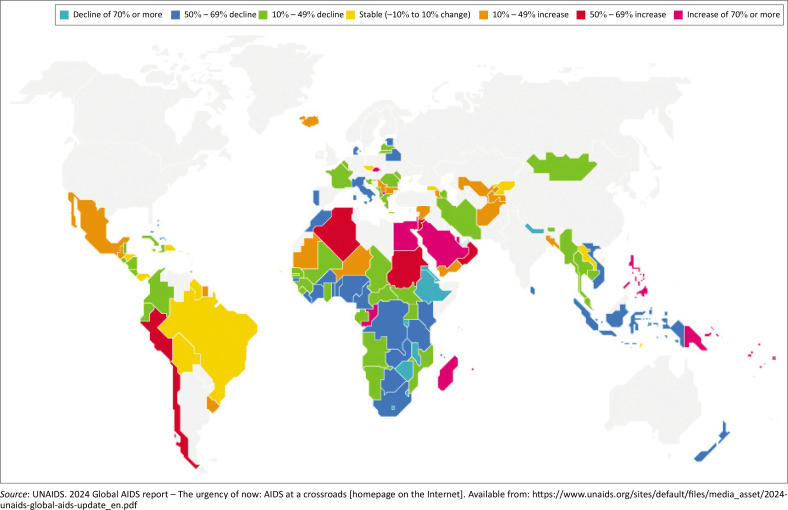
Changes in new human immnodeficieny virus infection, 2010–2022.

As part of the global financial investments to respond to the HIV and AIDS epidemic, the formation of ‘the Global Fund’ and its investment to defeat HIV and AIDS, TB, and malaria have been remarkable.^10^ The Abuja Declaration has supported the creation of the Global AIDS Fund by the donor community to enhance the operationalisation of HIV and AIDS, TB, and malaria control action plans, including increased access to antiretroviral therapies.^1^ Accordingly, after the Abuja Declaration, the Global Fund was created and operationalised in 2002 to confront these infectious diseases. Since then, the Global Fund has invested more than $60 billion to fight against these deadly diseases, of which $27.8bn (46.3%) was invested in HIV response. The Global Fund contributes 20% of the financing required for the HIV and AIDS response globally.^10^ Moreover, the formation of the United States (US) President’s Emergency Plan for AIDS Relief (PEPFAR) in 2002 was also instrumental in substantially financing the HIV and AIDS response in Africa. Since its inception, PEPFAR has invested more than $100bn to respond to HIV and AIDS globally – contributing to millions of lives saved.^11^ However, because of changes in priorities and political commitments, mainly as a result of the COVID-19 pandemic and wars, the gap in financing for the HIV and AIDS response is widening (Figure 2). For instance, in 2022, $20.8bn was available for HIV programmes in low- and middle-income countries (LMICs), 2.6% less than in 2021. Notably, 60% of the HIV and AIDS response was financed by domestic financing sources.^2^ Moreover, the recent United States Government (USG) decision to freeze funds will impact the HIV programme in Africa. The USG decided to stop PEPFAR’s support to South Africa and cut funding for several HIV prevention and control initiatives being implemented in African countries, underscoring the urgent need for self-reliance and innovative domestic funding to ensure continuity of care and treatment for patients with HIV. The Africa Centres for Disease Control and Prevention (Africa CDC) proposed innovative financing mechanisms for countries to implement to address the gaps in financing. As a short-term solution, the countries affected by the USG decision should work on shuffling and reprogramming their available resources towards essential life-saving HIV and AIDS programmes to ensure continuity of care. As a long-term solution, even where funds are available from external sources, it is critical to enhance the capacity of domestic financing to ensure the sustainability of HIV and AIDS response programmes.^12,13^

## Programmatic success – Decline in the burden of human immunodeficiency

Two decades ago, more than 2.5m people were acquiring HIV infection, and AIDS was claiming more than 2m lives every year.^2^ Access to life-saving prevention, care, and support interventions was highly limited in Africa, where the burden of the disease was the highest. However, the HIV and AIDS response, accompanied by ground-breaking innovations and investments such as antiretroviral therapies (ARTs), efficient screening and diagnostic tools, and innovative prevention and control strategies, has resulted in remarkable achievements and a significant decline in the burden of the disease.^14,15^

According to the UNAIDS 2023 report, in 2022, 25.6m (21.6m – 30.0m) people were living with HIV and AIDS (65.6% of the global estimate), 660 000 (480 000 – 920 000) became newly infected (50.8% of the global estimate), and 380 000 (296 000 – 530 000) died from AIDS-related illnesses in sub-Saharan Africa (SSA). In 2022, five SSA countries (Botswana, Eswatini, Rwanda, Tanzania, and Zimbabwe) achieved the 95-95-95 target, and eight other SSA countries (Kenya, Malawi, Namibia, Lesotho, Zambia, Uganda, Burundi, and Togo) were near to achieving the 95-95-95 target. The ‘Treat All’ initiative, rapid initiation of ART, and use of improved ART drugs (e.g., dolutegravir) substantially contributed to the improved treatment outcomes.^2^ A recent phase 3 clinical trial finding on the safety and effectiveness of lenacapavir has been a breakthrough for pre-exposure prophylaxis against HIV infection. The Africa CDC welcomes this innovation while encouraging further research to ensure the safety and effectiveness of the drug in other groups of the population.

Since 2001, Africa has made substantial progress towards HIV and AIDS control. In Eastern and Southern Africa, where the HIV burden and impacts are significant, 59% and 57% reductions in HIV new infections and AIDS-related deaths, respectively, were achieved in 2023 as compared to the magnitude in 2010. In 2023, 93% (75% – 98%) of people living with HIV knew their status, 83% (68% – 96%) of people living with HIV were on treatment, and 78% (72% – 86%) had viral suppression in Eastern and Southern Africa. Similar trends and progress have been made in the Central and Western Africa region, with 46% and 55% reductions in HIV new infections and AIDS-related deaths, respectively, achieved since 2010 as compared to the magnitude in 2010. In 2023, 81% (62% – 97%) of people living with HIV knew their status, 76% (59% – 92%) of people living with HIV were on treatment, and 70% (61% – 81%) had viral suppression in Central and Western Africa. However, a rise in new HIV infections was seen in Northern Africa (Figure 2).^2^

## Challenges and way forward

Despite its impressive accomplishments, innovations, and success stories, Africa has yet to overcome several challenges. The gap in financing for HIV and AIDS response is widening, particularly after the COVID-19 pandemic. The decision of the USG to freeze funds will also further widen funding gaps for HIV programmes. Achievements of HIV and AIDS control have exposed inequities, where adolescent girls and young women, children, and key populations (e.g., commercial sex workers), were left behind in accessing essential HIV and AIDS response measures.

For the sustained response to HIV and AIDS, increased domestic financing is essential and the way to go. It might be possible to attain the 95-95-95 UNAIDS target, but the long-term sustainability of the targets might be challenging. On the other hand, improvement in the HIV programme has implications for the health system as it increases older people receiving care and treatment and potentially complications related to the ART drugs. The health system needs to be prepared to accommodate the needs of this growing population through the integration of non-communicable diseases (NCDs) and mental healthcare and treatment into the HIV response system.

Although significant progress has been made towards the epidemic control of HIV and AIDS since the Abuja Declaration, the burden of the disease is still high in Africa – underscoring the need for concerted actions to address the gaps in response and pave the way towards ending AIDS as a public health threat by 2030. As we are near the 2030 landmark, revitalisation of the commitments made in the Abuja Declaration is essential. African countries must increase their domestic resources to address the inequities and improve access to essential HIV and AIDS response interventions, particularly for adolescent girls and young women, children, and vulnerable populations. Revitalisation of sex education, social protection, and revisiting in-country laws that negatively impact the response efforts are more essential than ever before. There is a clear need for rededication of political and leadership will and commitment as we envision epidemic control of HIV and AIDS by 2030. Countries need to develop an action-oriented, targeted, and all-inclusive roadmap for HIV and AIDS epidemic control by 2030. This roadmap must be accompanied by domestic innovative financing mechanisms, such as mobilising resources from the private sectors, local philanthropists, and community mobilisation initiatives.
